# Bidirectional relationship of problematic Internet use with hyperactivity/inattention and depressive symptoms in adolescents: a population-based cohort study

**DOI:** 10.1007/s00787-021-01808-4

**Published:** 2021-05-22

**Authors:** Masaya Morita, Shuntaro Ando, Tomoki Kiyono, Ryo Morishima, Tomoko Yagi, Sho Kanata, Shinya Fujikawa, Syudo Yamasaki, Atsushi Nishida, Kiyoto Kasai

**Affiliations:** 1 Department of Neuropsychiatry, The University of Tokyo Hospital, Tokyo, Japan; 2 Department of Neuropsychiatry, Graduate School of Medicine, The University of Tokyo, Tokyo, Japan; 3 Department of Psychiatry, Teikyo University School of Medicine, Tokyo, Japan; 4 Department of Psychiatry and Behavioural Sciences, Tokyo Metropolitan Institute of Medical Science, Tokyo, Japan

**Keywords:** Problematic Internet use, Internet addiction, Hyperactivity/inattention, Adolescent, Cohort study

## Abstract

Problematic Internet use (PIU), hyperactivity/inattention, and depressive symptoms are comorbid problems in adolescence, but the causal relationships among these issues are unclear. To assess the relationships among PIU, hyperactivity/inattention, and depressive symptoms in adolescents in the general population. This longitudinal cohort study used data from the Tokyo Teen Cohort study in Tokyo, Japan, for two years between October 2012 and January 2015. Of the 3171 pairs of children and parents, 3007 pairs continued to participate in the second wave of the Tokyo Teen Cohort study. A total of 3007 children were included in the analysis (mean [standard deviation] age, 9.7 [0.4] years; 1418 women [47.2%]. Cross-lagged panel analysis revealed that PIU at timepoint 1 was significantly associated with hyperactivity/inattention at timepoint 2 (*β* = 0.03; 95% confidence interval (CI) 0.01–0.06), and hyperactivity/inattention at timepoint 1 was also significantly associated with PIU at timepoint 2 (*β* = 0.07; 95% CI 0.04–0.10), even after adjustments were made for depressive symptoms. Furthermore, PIU at timepoint 1 was significantly associated with depressive symptoms at timepoint 2 (*β* = 0.05; 95% CI 0.01–0.12), and depressive symptoms at timepoint 1 were also significantly associated with PIU at timepoint 2 (*β* = 0.05; 95% CI 0.02–0.07), even after adjustments were made for hyperactivity/inattention. These results support the bidirectional relationships among PIU, hyperactivity/inattention, and depressive symptoms. PIU may be a target to improve hyperactivity/inattention and depressive symptoms in adolescents.

## Introduction

Problematic Internet use (PIU), which includes Internet addiction and compulsive Internet use, is characterized by an inability to control Internet use [1]. Recently, PIU has been a common problem among adolescents, with a prevalence of 7.9–16.0% [2, 3]. While Internet use is one of the most important methods for obtaining information about daily life and school study for adolescents, PIU is associated with negative behavioral and psychological outcomes in adolescents [4–7]. A typical behavioral feature of adolescents with PIU is hyperactivity/inattention (83.3%), and common co-occurrent psychopathology is depression (30%) [8]. The relationship between PIU and behavioral/psychological symptoms has been examined, but it is unclear whether the longitudinal relationship of each symptom is unidirectional or bidirectional.

With regard to the relationship between PIU and hyperactivity/inattention, there was evidence of only one direction of causality. A previous longitudinal study reported that attention-deficit hyperactivity disorder (ADHD) predicted the occurrence of PIU [9]. Although digital media use is associated with the risk of ADHD symptoms [10] and PIU was suggested to exacerbate ADHD symptoms in a review [11], there was no study that investigated whether PIU increased ADHD symptoms.

Regarding the relationship between PIU and depressive symptoms, there has been evidence of a bidirectional relationship, but these studies had certain limitations. A study showed that PIU was associated with elevated depression [12]. Additionally, a recent study has reported that screen time is associated with elevated depressive symptoms [13]. Furthermore, a previous study revealed a bidirectional relationship between PIU and depression [14]. However, these studies had several limitations, such as school-based sampling, a short follow-up period, assessing PIU by children’s self-report, and a lack of hyperactivity/inattention as a confounding factor [14].

Given the high prevalence of PIU and its comorbid hyperactivity/inattention and depression, it is required to examine the causal relationships between these issues. Therefore, we aimed to investigate the associations among PIU, hyperactivity/inattention, and depressive symptoms in adolescents using data from a longitudinal population-based cohort study. Since hyperactivity/inattention and depression affect each other [15], we hypothesized that there are bidirectional relationships among PIU, hyperactivity/inattention, and depressive symptoms. We used a cross-lagged panel analysis which enabled examination of the possibility that two variables influence each other in both directions [16].

## Methods

### Study design

This study used data from the Tokyo Teen Cohort study (TTC), a population-based longitudinal cohort survey [17]. The TTC is a multidisciplinary study aiming to investigate adolescent health and development. We recruited children from the participants in the Tokyo Early Adolescence Survey (T-EAS), which was a cross-sectional survey on the psychological and physical development of adolescents in the general population [18]. The participants were sampled by using the resident register in three municipalities in the metropolitan area of Tokyo, Japan: Setagaya-ku, Mitaka-shi, and Chofu-shi. Eligible participants were born between September 1st, 2002, and August 31st, 2004. The survey was conducted between October 2012 and January 2015. We treated the T-EAS data as the first wave data of the TTC (timepoint 1; T1). We sent letters of invitation to participants around their 10th birthday. After the letter was sent, trained interviewers visited potential participants’ homes. The survey was conducted during two home visits. On the first visit, written informed consent was obtained from the primary parent, and assent was obtained from the child. Participants were asked to complete the questionnaires at home before the second visit. During the second visit, both the child and the primary parent were asked to complete the self-report questionnaires separately. The questionnaires were placed in envelopes by the respondents immediately after completion. In addition, the primary parent completed a semistructured interview. All data were anonymized using the study IDs, and the lists for matching were kept strictly confidential. We conducted the second wave of the TTC study between July 2014 and January 2017, when the children were 12 years old (timepoint 2; T2). The second wave was implemented by means of the same procedure used in the first wave. The TTC was conducted by three research institutes: Tokyo Metropolitan Institute of Medical Science, the University of Tokyo and SOKENDAI (The Graduate University for Advanced Studies). This study was approved by the ethics committees of these three institutes.

### Participants

**Fig. 1:**
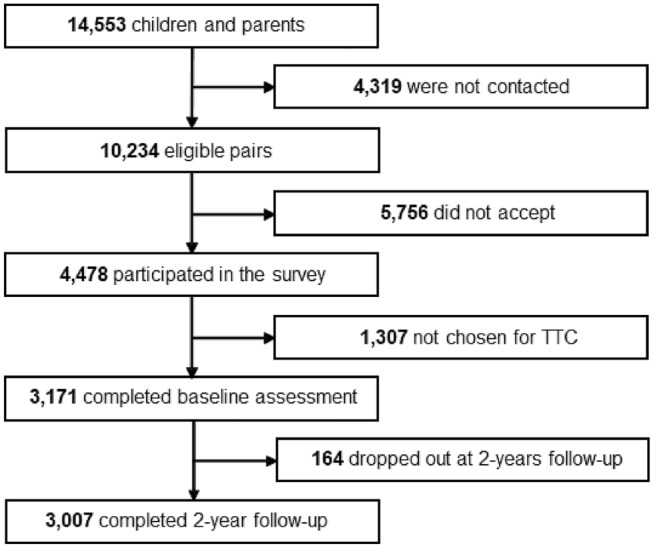
Participants in the study of problematic Internet use and hyperactivity/inattention and/or depressive symptoms

Among the 14,553 children and primary parents randomly chosen from the resident register, 4319 pairs could not be contacted. Of the 10,234 accessible pairs, a total of 4478 children and their primary parents participated in the T-EAS (response rate: 43.8%). An oversampling method was used in the collection of TTC participants, considering the low follow-up rate of families with low household income [17]. Hence, 3171 participants in the T-EAS were recruited for the TTC. Of the 3171 pairs of children and parents, 3007 pairs continued to participate in the second wave of the TTC (follow-up rate: 94.8%). We analyzed the data obtained from 3007 pairs who participated in the second wave (Fig. 1).

### Measures

Primary parents answered self-administered questionnaires that included questions about their children’s PIU, children’s hyperactivity/inattention, household income, father’s educational background, mother’s educational background, screen time and other variables, such as children’s age in months and sex. Children answered self-administered questionnaires that included questions about their depressive symptoms.

### PIU

We assessed PIU with the modified scale of the Compulsive Internet Use Scale [19]. The Japanese version of the CIUS was validated [20]. We modified each self-administered question to create a question for parents. The following questions were used to assess PIU: “Do others (e.g., teachers, parents and friends) say your child should use the electric devices such as the Internet or mobile phone, less?”; “Do you feel your child should decrease the amount of time you spend using the electric devices such as the Internet or mobile phone?”; “Does your child find it difficult to stop using the electric devices, such as the Internet or mobile phones, once he/she starts?”; “Is your child short of sleep due to being on his/her phone or the Internet late at night?”; “Does your child neglect his/her daily obligations (e.g., homework or housework) because he/she prefer to go on the Internet or use mobile phones?”; “Does your child feel frustrated or irritated when he/she cannot use the Internet or mobile phone?”; “Does your child use the electric devices, such as the Internet or mobile phones, to escape from his/her sorrows or get relief from negative feelings?”; “Does your child’s use of the electric devices, such as the Internet or mobile phones, cause problems with friends or family?”; “Does your child prefer to use the Internet instead of spending time with others?”; and “Do others say your child spends too much time on the Internet?” The parents were asked to choose one from the following four answer options: never, 0; sometimes, 1; often, 2; and don’t know, 3. We created a continuous variable for PIU by summing 2 points for "often" and 1 point for "sometimes"; the total scores for this variable ranged from 0 to 20. Cronbach’s alpha for the PIU score was 0.87 at T1 and 0.87 at T2.

### Hyperactivity/inattention

We used the validated Japanese version of the Strengths and Difficulties Questionnaire (SDQ), a widely used parent-reported questionnaire, to evaluate the behavioral problems of the adolescents [21]. The test–retest reliability of the measure was reported as good (hyperactivity/inattention *ρ* = 0.70, 0.84) [22]. The SDQ is shown to be a useful tool for assessing ADHD symptoms [23]. To evaluate adolescents’ hyperactivity/inattention, we used a subscale of the SDQ that includes five questions rated on a 3-point Likert scale: not true—0; somewhat true—1; and absolutely true—2. These scores are summed into a hyperactivity/inattention score, which has a possible score range from 0 to 10. Higher scores indicate more hyperactivity/inattention [24].

### Depressive symptoms

The Short Mood and Feelings Questionnaire (SMFQ) was used for the evaluation of adolescents’ depressive symptoms. The SMFQ includes 13 self-administered questions rated on a 3-point Likert scale: not true—0; somewhat true—1; and absolutely true—2. The total score ranges from 0 to 26 and can be used to measure the severity of depressive symptoms [25].

### Covariates

We selected the following variables as potential confounders due to their association with PIU, hyperactivity/inattention and depression in previous studies [26] age in months, sex, household income, father’s and mother’s educational background as indicators of family socioeconomic status, and screen time. Parents reported household income (1 [0–990,000 yen] to 11 [≥ 10 million yen]). Parents’ educational background was categorized into six groups (1 [graduated junior high school], 2 [dropped out of high school], 3 [graduated high school], 4 [2-year college], 5 [4-year university], and 6 [graduate school]). For screen time, parents evaluated the length of time that their children watched TV programs and movies on a weekday, including on personal computers and mobile devices (1 [none], 2 [≤ 1 h], 3 [1–2 h], 4 [2–3 h], 5 [3–5 h], 6 [5–7 h], and 7 [≥ 7 h]), and the length of time that their children played games on personal computers and consumer games on a weekday (1 [none], 2 [≤ 1 h], 3 [1–2 h], 4 [2–3 h], 5 [3–5 h], 6 [5–7 h], and 7 [≥ 7 h]).

### Statistical analyses

**Fig. 2:**
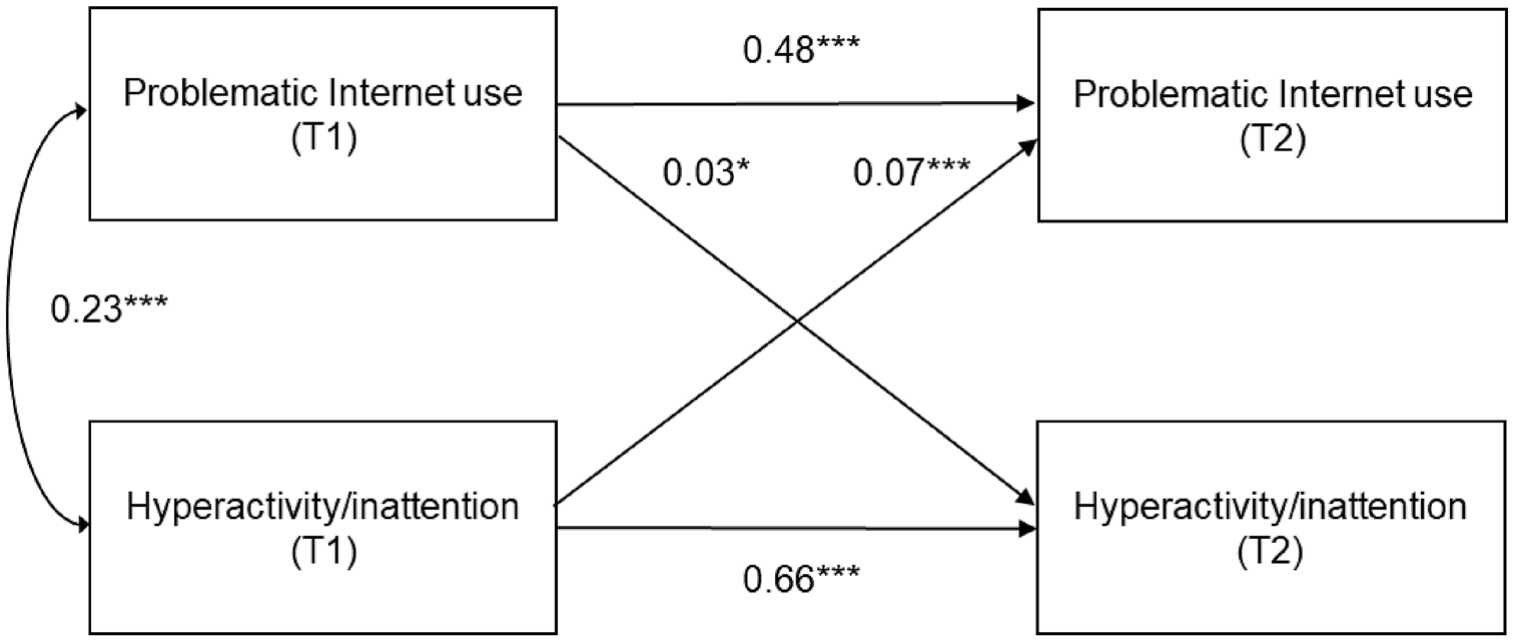
Cross-lagged model of associations between problematic Internet use and hyperactivity/inattention. *χ*^2^ = 14.23, *df* = 10, *p* = 0.16, Comparative Fit Index = 0.99, Tucker–Lewis Index = 0.99, root mean square error of approximation = 0.012. Standardized coefficients were adjusted for sex, age in months, father's educational background, mother's educational background, screen time, and depressive symptoms. *p < 0.05, ***p < 0.001

To identify causality, we performed cross-lagged panel analysis, which was useful to examine the bidirectional relationships among variables simultaneously over time. We created a model using full information maximum likelihood estimation to estimate the effects of missing data [27]. Model fit indices were determined by the comparative fit index (CFI), the Tucker-Lewis index (TLI), and the root mean square error of approximation (RMSEA). CFI and TLI values greater than 0.95 were considered a good fit, and RMSEA values of less than 0.06 were considered a good fit [28]. The structure of the cross-lagged model for PIU and hyperactivity/inattention is shown in Fig. 1 and that for PIU and depressive symptoms is shown in Fig. 2. These models included sex, age in months, household income, father's educational background, mother's educational background, and screen time as covariates. We examined multicollinearity of covariates by using VIF (Variance Inflation Factor). Cross-lagged panel analyses were conducted in the Statistical Package for Social Sciences (SPSS) Amos, version 7.0. The other analyses were tested in SPSS, version 25 for Windows (SPSS Inc., Tokyo, Japan). A *p* value < 0.05 was considered statistically significant.

## Results

### Descriptive analyses

Table 1 shows the descriptive statistics for the participants in this study. Of the 3171 participants at T1, a total of 3007 participants completed all questionnaires at T2. In the analytic sample, 47.2% at T1 were girls. The mean age of the participants at T1 was 9.7 years old (standard deviation; SD 0.4 years) (Table 1) which met the criteria of adolescents [29]. The Cronbach’s alpha of PIU, hyperactivity/inattention, and depressive symptoms were ≥ 0.70, which satisfied the validity criteria [30]. There was no multicollinearity between the variables of household income, father's educational background, and mother's educational background.

**Table 1:** Descriptive statistics of the participants in this study (*N* = 3007)

	Value	Missing, *n* (%)	Cronbach's *α*
Girls, *n* (SD)	1418 (47.2)	0 (0.0)	
Age, y, mean (SD)	9.7 (0.4)	4 (0.1)	
Household income, no. (%), 10,000 yen		113 (3.8)	
≤ 500	582 (19.4)		
500–999	1449 (48.1)		
≥ 1000	866 (28.8)		
Father's educational background, no. (%)		138 (4.6)	
Graduated junior high or high school	516 (17.2)		
2-year or 4-year university	1998 (66.5)		
Graduate university	355 (11.8)		
Mother's educational background, no. (%)		23 (0.7)	
Graduated junior high or high school	498 (16.6)		
2-year or 4-year university	2383 (79.3)		
Graduate university	103 (3.4)		
Weekday screen time for TV, h (%)		22 (0.7)	
≤ 1	705 (23.4)		
1–3	1870 (62.2)		
3–5	364 (12.1)		
≥ 5	46 (1.5)		
Weekday screen time for games, h (%)		31 (1.0)	
≤ 1	2149 (71.5)		
1–3	763 (25.4)		
3–5	54 (1.8)		
≥ 5	10 (0.3)		
Problematic internet use score			
At T1, mean (SD)	2.7 (3.5)	64 (2.1)	0.87
At T2, mean (SD)	3.9 (4.2)	38 (1.2)	0.87
SDQ hyperactivity/inattention score			
At T1, mean (SD)	3.0 (2.2)	10 (0.3)	0.76
At T2, mean (SD)	2.7 (2.1)	14 (0.4)	0.75
SMFQ total score			
At T1, mean (SD)	4.7 (4.6)	45 (1.5)	0.86
At T2, mean (SD)	3.9 (4.5)	490 (16.3)	0.87

*SD* standard deviation, *T1* timepoint 1, *T2* timepoint 2, *SDQ* Strength and Difficulties Questionnaire, *SMFQ* the Short Mood and Feelings Questionnaire

Table 2 shows the correlation between the confounding variables and the examined variables at T1. The cross-sectional correlation between PIU and hyperactivity/inattention was 0.23 at T1, and 0.27 at T2. The cross-sectional correlation between PIU and depression was 0.12 at T1, and 0.16 at T2.

**Table 2:** Correlation matrix of variables in the present study

	1	2	3	4	5	6	7	8	9	10
1. Problematic Internet use	–									
2. SDQ hyperactivity/inattention	0.23^***^	–								
3. SMFQ	0.12^***^	0.25^***^	–							
4. Sex	− 0.17^***^	− 0.19^***^	− 0.09^***^	–						
5. Age in month	0.04	− 0.01	− 0.01	− 0.02	–					
6. Father's educational background	− 0.04	− 0.14^***^	− 0.09^***^	0.02	− 0.01	–				
7. Mother's educational background	− 0.04^*^	− 0.11^***^	− 0.07^***^	0.03	− 0.02	0.4^***^	–			
8. Household income	− 0.06^**^	− 0.13^***^	− 0.09^***^	0.02	0.02	0.42^***^	0.33^***^	–		
9. Weekday screen time for TV	0.27^***^	0.14^***^	0.11^***^	− 0.05^**^	0.03	− 0.25^***^	− 0.27^***^	− 0.22^***^	–	
10. Weekday screen time for games	0.4^***^	0.16^***^	0.13^***^	− 0.19^***^	0.04^*^	− 0.23^***^	− 0.22^***^	0.19^***^	0.49^***^	–

*p < 0.05, **p < 0.01, ***p < 0.001

*SDQ* Strength and Difficulties Questionnaire, *SMFQ* the Short Mood and Feelings Questionnaire

Figure 2 shows the association between PIU and hyperactivity/inattention. In the cross-lagged panel analysis, PIU at T1 was significantly associated with hyperactivity/inattention at T2 (*β* = 0.03, 95% confidence interval (CI) 0.01–0.06) in the adjusted model. Hyperactivity/inattention at T1 was significantly associated with PIU at T2 (*β* = 0.07, 95% CI 0.04–0.10). Fit indices revealed that the model was a good fit to the observed data (*χ*^2^ = 14.23; *p* = 0.16; CFI = 0.99; TLI = 0.99; RMSEA = 0.012). The coefficient of determination for PIU at T2 was 0.33, and that for hyperactivity/inattention at T2 was 0.51.

**Fig. 3:**
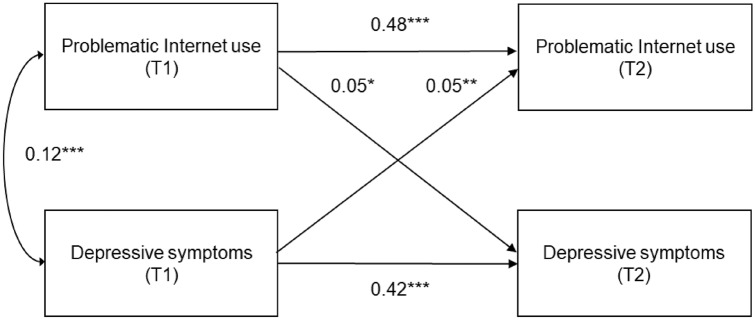
Cross-lagged model of associations between problematic Internet use and depressive symptoms. *χ*^2^ = 14.87, *df* = 10, *p* = 0.14, Comparative Fit Index = 0.99, Tucker–Lewis Index = 0.99, root mean square error of approximation = 0.013. Standardized coefficients were adjusted for sex, age in months, father's educational background, mother's educational background, screen time, and hyperactivity/inattention. *p < 0.05, **p < 0.01, ***p < 0.001

Figure 3 shows the association between PIU and depressive symptoms. PIU at T1 was significantly associated with depressive symptoms at T2 (*β* = 0.05; 95% CI 0.01–0.12). Additionally, depressive symptoms at T1 were significantly associated with PIU at T2 (*β* = 0.05; 95% CI 0.02–0.07) in the adjusted model. Fit indices revealed that the model was a good fit to the observed data (*χ*^2^ = 14.87; *p* = 0.14; CFI = 0.99; TLI = 0.99; RMSEA = 0.013). The coefficient of determination for PIU at T2 was 0.33, and that for depressive symptoms at T2 was 0.20.

## Discussion

To the best of our knowledge, this was the first study that revealed a bidirectional relationship between PIU and hyperactivity/inattention in adolescents in the general population even after adjustments were made for depressive symptoms. Furthermore, it became clear that there was a bidirectional relationship between PIU and depressive symptoms even after adjustments were made for hyperactivity/inattention. The strengths of this study included the follow-up of these adolescents for two years, the objective assessment of PIU using parent report, and the adjustment for screen time as a covariate.

The results of this study were consistent with the findings from previous studies showing that hyperactivity/inattention predicted PIU [9]. Additionally, this study showed the reverse finding that PIU exacerbated hyperactivity/inattention. In previous studies, a higher frequency of digital media use predicted ADHD symptoms, and screen time predicted poor child development [10, 13, 31]. PIU is a construct that focuses not only on the length of time and frequency of Internet use but also on the factor of addiction, such as salience (i.e., becoming the most important activity in a person’s life), withdrawal symptoms (i.e., feeling agitated and irritable when the Internet is inaccessible), mood modification (i.e., feeling moody when unable to use the Internet), tolerance (i.e., needing to increase amounts of the particular activity to get the former effects), conflict (i.e., to conflict in the addictive life with personal relationships, educational lives and other social activities), and relapse (i.e., unsuccessful efforts to stop or decrease the use of the Internet) [32, 33]. In our study, screen time did not predict hyperactivity/inattention. Therefore, it is thought that the addictive aspects of Internet use exacerbate hyperactivity/inattention.

There are several possible explanations for the direction in which PIU can predict hyperactivity/inattention. First, children with PIU may not engage in other activities when they immerse themselves in the Internet. Consequently, children have fewer opportunities to play sports or engage in artistic activities, which are said to aid in the development of behavioral self-control [11]. Children with a shortage of self-control may experience worsened ADHD symptoms. Second, obtaining rewards quickly and gaining easy access to the Internet may weaken attention and behavioral control in the real world [34]. Compared to the Internet environment, real activities require the investment of time to obtain rewards; as such, children can become restless and distracted by not being rewarded. Third, problematic Internet users may regard Internet use as their highest priority. They may become sensitive to personal computers or mobile phone notifications of changes on the Internet. Frequent distractions can interfere with ongoing attention. Continuous exposure to a distracted environment can result in the inability to acquire sustained attention [10, 35].

The bidirectional relationship between PIU and hyperactivity/inattention may lead to a vicious circle of children's behavioral and psychological aspects. Children with hyperactivity/inattention have two core symptoms: ‘being easily bored’ and ‘having an aversion for delayed rewards’ [36]. They may have difficulty adapting to the real world, where it is difficult to obtain short-term rewards; instead, they may immerse themselves in the Internet world, where short-term aspirations can be fulfilled quickly. As mentioned above, Internet immersion is likely to cause an increase in hyperactivity/inattention symptoms. Therefore, children with hyperactivity/inattention symptoms may tend to be in a vicious cycle of Internet immersion, where they tend to become more hyperactive/inattentive. In recent years, the existence of late-onset ADHD has become apparent [37]. There may be a possibility that late-onset ADHD may be exacerbated by PIU.

This study also revealed a bidirectional association between PIU and depressive symptoms, even after adjustments were made for hyperactivity/inattention and screen time. To overcome the limitations of the previous studies that evaluated PIU only by participant self-report [9, 12], we evaluated PIU by parental report. The two-way relationship between PIU and depressive symptoms may lead to another vicious cycle affecting adolescents’ mental health. Children with depressive symptoms are more likely to become immersed in the Internet environment for the purposes of avoiding reality and seeking relief. Depressive children will tend to use the Internet problematically to escape from negative emotions [38]. Immersion in the Internet environment is likely to lead to decreased daily activities, excessive daytime sleep, exposure to social network slander, and online bullying [39]. Therefore, PIU in children may lead to the aggravation of depressive symptoms. Similarly, screen time did not predict depressive symptoms in this study. For this reason, the addictive aspects of Internet use may worsen depressive symptoms more than the addictive aspects of screen time.

Since PIU had a bidirectional relationship with both hyperactivity/inattention and depressive symptoms, one symptom may increase the possibility of the future emergence/deterioration of the other symptom. Therefore, caregivers of children need to check if there is another symptom underlying the main symptom, and they should also pay attention to the possibility of another symptom appearing. It can also be assumed that improving one symptom may improve the other symptom and break the vicious cycle [40]. It is possible that hyperactivity/inattention or depressive symptoms may be additional therapeutic targets for PIU and vice versa. From these, we can identify two public health implications. First, it is important to educate adolescents about appropriate Internet use to prevent PI. Preventing PIU in adolescents may help maintain their mental health by preventing subsequent hyperactivity/inattention and depression. Second, it is important to keep in mind that adolescents with PIU may have prior hyperactivity/inattention and depression. Identifying and treating hyperactivity/inattention and depression that preceded PIU may be expected to improve PIU in adolescents.

With regard to limitations, the standardized estimates in this study were low. Future research is needed to determine if there is a definite causal relationship. Second, as participants of this study consist of only Asian people, it is necessary to evaluate our findings in other ethnic groups in the future. Third, we did not assess participants’ reasons for using the Internet (i.e., studying, homework, social networking sites, Internet gaming, or porn sites). Future studies will be needed to determine which usage has the most impact. Fourth, we did not assess other potential confounders, such as social anxiety disorder, hostility, or parents’ Internet use. Finally, our study period was only two years. Therefore, it is necessary to continue tracking these relationships in the future.

## Conclusions

A bidirectional relationship between PIU and hyperactivity/inattention was observed in the two-year follow-up of 3007 10-year-old children in the general population. PIU increased hyperactivity/inattention two years later, and hyperactivity/inattention exacerbated PIU two years later. Similarly, a bidirectional relationship was found between PIU and depressive symptoms. Future research should focus on whether interventions with PIU would improve hyperactivity/inattention and depressive symptoms in adolescents. Similarly, interventions with hyperactivity/inattention and depressive symptoms might normalize Internet use.

## Data Availability

The data that support the findings of this study are available from Tokyo Teen Cohort study but restrictions apply to the availability of these data, which were used under license for the current study, and so are not publicly available. Data are, however, available from the authors upon reasonable request and with permission of Tokyo Teen Cohort study.
